# The *Schizosaccharomyces pombe* Hsp104 Disaggregase Is Unable to Propagate the [*PSI* ^+^] Prion

**DOI:** 10.1371/journal.pone.0006939

**Published:** 2009-09-11

**Authors:** Patrick Sénéchal, Geneviève Arseneault, Alexandre Leroux, Susan Lindquist, Luis A. Rokeach

**Affiliations:** 1 Department of Biochemistry, Université de Montréal, Montréal, Québec, Canada; 2 Whithead Institute for Biomedical Research and Howard Hughes Medical Institute, Cambridge Center, Cambridge, Massachusetts, United States of America; Baylor College of Medicine, United States of America

## Abstract

The molecular chaperone Hsp104 is a crucial factor in the acquisition of thermotolerance in yeast. Under stress conditions, the disaggregase activity of Hsp104 facilitates the reactivation of misfolded proteins. Hsp104 is also involved in the propagation of fungal prions. For instance, the well-characterized [*PSI^+^*] prion of *Saccharomyces cerevisiae* does not propagate in *Δhsp104* cells or in cells overexpressing Hsp104. In this study, we characterized the functional homolog of Hsp104 from *Schizosaccharomyces pombe* (Sp_Hsp104). As its *S. cerevisiae* counterpart, *Sp_hsp104^+^* is heat-inducible and required for thermotolerance in *S. pombe*. Sp_Hsp104 displays low disaggregase activity and cannot propagate the [*PSI^+^*] prion in *S. cerevisiae*. When overexpressed in *S. cerevisiae*, Sp_Hsp104 confers thermotolerance to *Δhsp104* cells and reactivates heat-aggregated proteins. However, overexpression of Sp_Hsp104 does not propagate nor eliminate [*PSI^+^*]. Strikingly, [*PSI^+^*] was cured by overexpression of a chimeric chaperone bearing the C-terminal domain (CTD) of the *S. cerevisiae* Hsp104 protein. Our study demonstrates that the ability to untangle aggregated proteins is conserved between the *S. pombe* and *S. cerevisiae* Hsp104 homologs, and points to a role of the CTD in the propagation of the *S. cerevisiae* [*PSI^+^*] prion.

## Introduction

Yeasts have the ability to survive a broad spectrum of stress conditions. When exposed to mild stresses, cells trigger an adaptive response to enhance their protection. This adaptation endows the cells with the ability to survive more severe stresses. In the case of heat shock, this adaptive response is called thermotolerance. Hsp104 is an AAA+ protein (ATPase associated with various activities) involved in the acquisition of thermotolerance in yeasts [1]. Unlike classical molecular chaperones that assist protein folding and prevent their aggregation, Hsp104 is a disaggregase that dissolves preformed protein aggregates [2]. The *Saccharomyces cerevisiae* Hsp104 protein contains two ATPase domains that are both involved in disaggregation. The first nucleotide-binding domain (NBD1) is crucial for substrate binding, while the second NBD2 domain is involved in the processing of protein aggregates and in the oligomerization of Hsp104 [3], [4], [5], [6]. The activities of the two NBDs are modulated by the middle (M) domain [7]. *In vitro* experiments demonstrated that Hsp104 binds and untangles aggregated proteins, releasing them in a folding-competent state [5], [8]. These observations are consistent with data obtained for ClpB, the bacterial homolog of Hsp104, suggesting that the mechanisms of protein disaggregation are well conserved across the ClpB/Hsp100 family [9], [10]. Although Hsp104 and ClpB are able to untangle protein aggregates by themselves, collaboration with the Hsp40 and Hsp70 families of molecular chaperones greatly enhances the disaggregating activities of both Hsp104 and ClpB [11], [12], [13], [14]. Interestingly, this chaperone interaction is species-specific since bacterial chaperones cannot collaborate efficiently with Hsp104 [11], [13].

Apart from its role as a disaggregase during heat stress, Hsp104 is also essential for the propagation of several prions in *S. cerevisiae*. Prions are proteins that can adopt at least two conformations, one of which is self-replicative. The best characterized yeast prions are [*PSI^+^*], [*URE3*] and [*PIN^+^*] from the budding yeast. In *S. cerevisiae*, these prions form amyloid fibers, and cannot be propagated in cells devoid of Hsp104 [15], [16], [17], [18]. [*PSI^+^*] is the most studied yeast prion. [*PSI^+^*] results from the amyloidogenic conformational switch of the Sup35 protein, a factor involved in the fidelity of translation termination [19].

The role of Hsp104 in prion propagation is not completely understood. The current model suggest that prion fibers need to be shortened or cleaved by Hsp104 in order to be correctly transmitted to the mitotic progeny during cell division [20], [21]. This model is supported by *in vivo* experiments showing that a reduction of the quantity of Hsp104 results in an increase of the length of the [*PSI^+^*] fibrils as well as a reduction in their ability to be transmitted from to daughter cells [22], [23]. On the other hand, overexpression of Hsp104 eliminates (or “cures”) the [*PSI^+^*] prion [15]. Moreover, Hsp104 is able to fragment Sup35p amyloid fibers [24]. Interestingly, a number of studies showed that mutants of Hsp104 can differentially affect thermotolerance, prion propagation and prion curing [25], [26], [27]. The Hsp104 homolog of the yeast *Candida albicans* also propagates [*PSI^+^*] in *S. cerevisiae*, suggesting that this ability is conserved among yeast species [28].

Whereas protein folding has been extensively studied in budding yeast, less is known about the function of molecular chaperones in *Schizosaccharomyces pombe*. Some studies reported that the fission yeast can acquire thermotolerance [29], [30], but the involvement of a putative Hsp104 homolog in this process has not been confirmed so far. In this study, we characterized for the first time an *S. pombe* protein (Sp_Hsp104) showing a high level of sequence identity with the *S. cerevisiae* Hsp104 protein. Our results demonstrate that Sp_Hsp104 is a heat-inducible disaggregase and a crucial factor in the acquisition of thermotolerance in fission yeast. Heterologous expression of Sp_Hsp104 in *S. cerevisiae* confirmed that this protein is a functional homolog of Hsp104 for thermotolerance. However, unlike the budding yeast Hsp104, Sp_Hsp104 did not support the propagation of the [*PSI^+^*] prion under basal expression levels. Furthermore, overexpression of Sp_Hsp104 did not cure [*PSI^+^*]. Remarkably, a chimeric Sp_Hsp104 bearing the CTD of *S. cerevisiae* Hsp104 gained the ability to cure the [*PSI^+^*] prion. These observations suggest that the CTD region of Hsp104 plays an important role in the propagation of prions.

## Results

### *S. pombe* encodes a single Hsp104 homolog

**Figure 1:**
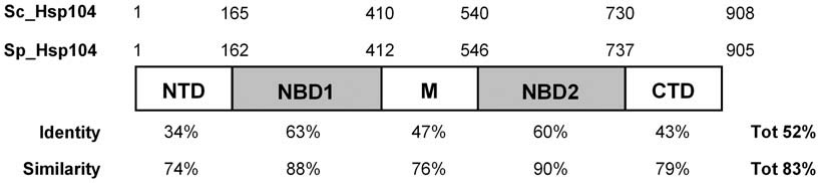
Sequences comparison between Hsp104 homologs. Schematic representation of the five domains of Hsp104 as described by Hung *et al.* (2006) [25]. Hsp104 has two nucleotide-binding domains (NBD1 and NBD2), which are well conserved among AAA+ proteins. These domains are separated by a middle (M) domain that is specific to the ClpB/Hsp100 subfamily. The N- and C-terminal domains (NTD and CTD) are the least conserved domains. The position of the first amino acid of each region is indicated above the representation of the *S. cerevisiae* and *S. pombe* Hsp104 homologs (Sc_Hsp104 and Sp_Hsp104, respectively). The percentage of identity and similarity between both Hsp104 homologs is indicated below each domain.

To identify a putative *S. pombe* homolog of Hsp104, we performed a protein-protein BLAST search (http://www.ncbi.nlm.nih.gov/BLAST/) against the *S. pombe* proteome. A single *S. pombe* ORF, SPBC16D10.08c [31], encoded a predicted polypeptide with more than 52% identity and 83% similarity with the *S. cerevisiae* Hsp104 protein (Figure 1). This predicted *S. pombe* protein was not functionally characterized before. However, genome-wide expression analyses demonstrated that the gene is expressed and that the protein is translated and localizes to both the cytoplasm and the nucleus [32], [33], [34], [35], [36]. We named the gene *hsp104^+^* according to the *S. pombe* terminology. However, to avoid any confusion with the *HSP104* gene from *S. cerevisiae* (*Sc_HSP104*), in this article the *hsp104* ^+^ gene will be referred to as *Sp_hsp104^+^*, and the synthesized protein Sp_Hsp104. Accordingly, proteins from *S. pombe* and *S. cerevisiae* will be referred to as Sp_Hsp104 and Sc_Hsp104, respectively.

Like all members of the ClpB/Hsp100 family, Sp_Hsp104 contains two putative nucleotide-binding domains (NBD1 and NBD2) that are well conserved between *S. pombe* and *S. cerevisiae* (63% and 60% amino acid identity, respectively). All the critical residues required for the ATPase activity of those domains are perfectly conserved between these two proteins [28]. The M domain, which is important for the disaggregase activity, shares 47% amino acid identity between Sp_Hsp104 and Sc_Hsp104 (Figure 1). The M domain of Sp_Hsp104 also possesses the typical leucine-zipper motifs, suggesting that this domain adopts a functional structure (not shown). Hence, the fission yeast homolog encoded by SPBC16D10.08c exhibits all the ClpB/Hsp100 characteristics required for the disaggregase activity.

### The *Sp_hsp104^+^* gene is required for thermotolerance in *S. pombe*

Sc_Hsp104 is a major factor involved in the acquisition of thermotolerance in the budding yeast [1]. Interestingly, it was shown in an *S. pombe* genome-wide microarray study that the SPBC16D10.08c gene (*Sp_hsp104^+^*) is overexpressed 54 fold by a mild heat shock of 15 minutes at 39°C [33]. This demonstrates that the expression of *Sp_hsp104^+^* is greatly enhanced by environmental stresses, similarly to *Sc_HSP104* [37], [38].

**Figure 2:**
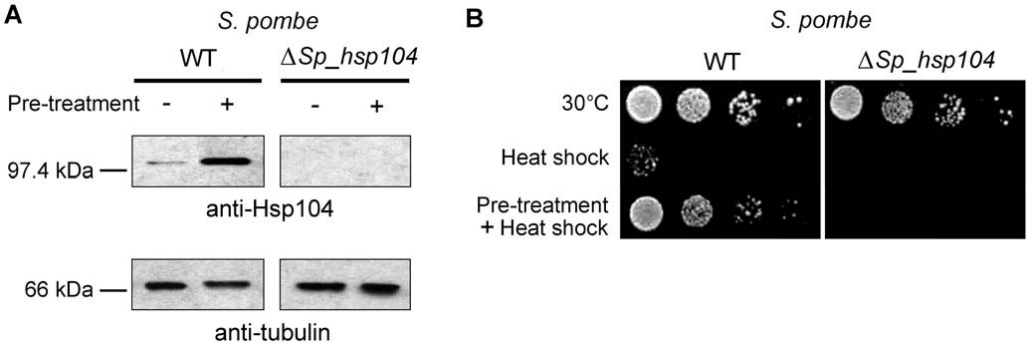
Sp_Hsp104 is required for thermotolerance in *S. pombe*. (A) The *Sp_hsp104^+^* gene is heat-inducible. WT (SP3220) and Δ*hsp104* (SP12422) *S. pombe* cells were treated or not for 1 hour at 37°C. Cell extracts were separated by SDS-PAGE, transferred onto a nitrocellulose membrane and immunoblotted with rabbit polyclonal antibodies against Sc_Hsp104 [6]. Immunoblot against tubulin is shown as a loading control. (B) The *Sp_hsp104^+^* gene is required for thermotolerance. WT (SP3220) and Δ*hsp104* (SP12422) *S. pombe* cells were submitted to a thermotolerance assay. Pre-treatment of 1 hour at 37°C was applied or not before heat shock at 50°C for 20 minutes. Cells were then briefly cooled on ice, serially diluted (10^−1^, 10^−2^, 10^−3^, 10^−4^), spotted on minimal media and incubated for 5 days at 30°C. Results are representative of three independent experiments.

To confirm that Sp_Hsp104 is heat-inducible, we carried out immunoblots in different conditions with polyclonal antibodies raised against the full-length Sc_Hsp104 protein [6]. These antibodies detected a ∼100 kDa protein in the wild-type fission yeast strain (SP3220), but no protein in the Δ*Sp_hsp104* strain (SP12422); thus confirming the specificity of the immunodetection (Figure 2A). Quantification of each band revealed that a 1-hour pre-treatment at 37°C increased the level of Sp_Hsp104 protein by ∼100-fold, as compared to unstressed cells (Figure 2A). Thus, like its *S. cerevisiae* counterpart, Sp_Hsp104 expression is induced by heat.

To investigate whether Sp_Hsp104 is able to confer protection against heat in fission yeast, we performed thermotolerance assays. As shown in Figure 2B, Δ*Sp_hsp104* cells did not show any significant growth defect when maintained at 30°C. Likewise, neither wild-type nor Δ*Sp_hp104* cells showed growth defects at 37°C (not shown). In contrast, most WT and Δ*Sp_hsp104* cells did not survive a severe heat shock of 20 minutes at 50°C. When WT cells were pre-treated at 37°C for 1 hour before being submitted to a severe heat shock, they acquired thermotolerance and survived (Figure 2B). However, Δ*Sp_hsp104* cells did not form colonies following heat shock at 50°C, even after pre-treatment at 37°C. This demonstrates that Sp_Hsp104 is required for thermotolerance in *S. pombe*.

### Sp_Hsp104 is a functional disaggregase in *S. cerevisiae*

**Figure 3:**
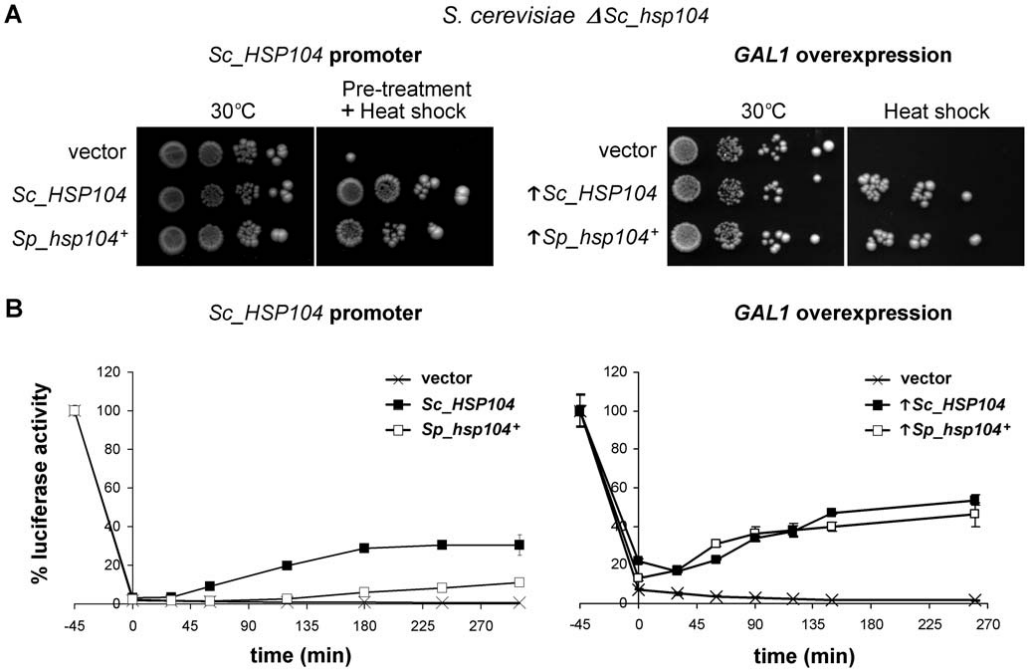
Sp*_*Hsp104 complements the knockout of *Sc_HSP104* in *S. cerevisiae*. (A) Heterologous complementation of thermotolerance. Δ*hsp104 S. cerevisiae* cells (SL303a) bearing an empty vector or a plasmid expressing either *Sc_HSP104* or *Sp_hsp104^+^* under the control of the *S. cerevisiae HSP104* promoter (left panel), or under the control of the *GAL1* overexpression promoter (right panel) were pre-treated or not for 1 h at 37°C before a heat shock of 20 minutes at 50°C. Cells were then briefly cooled on ice, serially diluted (10^−1^, 10^−2^, 10^−3^, 10^−4^), spotted on minimal media and incubated for 5 days at 30°C. Results are representative of three independent experiments. (B) Luciferase reactivation assay. Δ*hsp104 S. cerevisiae* cells (SL303a) expressing one of the Hsp104 homologs and bacterial luciferase were submitted to a heat shock of 46°C for 45 minutes to inactivate the protein. Reactivation was assessed by measuring luciferase activity at the indicated time points and reporting the value on the initial activity (100%). Reactivation curves of cells bearing an empty vector (X), expressing either *Sc_HSP104* (▪) or *Sp_hsp104^+^* (□) (left panel), or overexpressing (↑) these genes (right panel) are indicated. Each point is the mean±standard deviation of three independent measurements.

To verify that the *S. pombe* Hsp104 homolog exhibits disaggregase activity, the *Sp_hsp104^+^* gene was cloned into a centromeric plasmid in which expression is driven by the native promoter of *Sc_HSP104*. The resulting construct was transformed into *Δhsp104 S. cerevisiae* cells. Expression of *Sp_hsp104* ^+^ was confirmed by Western blotting using polyclonal anti-Hsp104 antibodies (Supplemental Figure S1A). To assess the ability of *Sp_hsp104^+^* to complement thermotolerance in *S. cerevisiae*, we performed serial-dilution assays. As shown in Figure 3A (left panel), heterologous expression of Sc_Hsp104 had no effect on growth at 30°C as compared to the endogenous Hsp104. As expected, exposing the cells to a severe heat shock at 50°C resulted in the death of most *Δhsp104 S. cerevisiae* cells (not shown). However, after a pre-treatment of 1 hour at 37°C, cells expressing either the fission yeast or the budding yeast Hsp104 survived as well as the untreated cells (Figure 3A, left panel). To verify if thermotolerance could also be achieved by constitutive overproduction of Sp_Hsp104, we cloned the *Sp_hsp104^+^* gene in a centromeric plasmid under the control of the *GAL1* promoter. The *GAL1* promoter increases gene expression 1000 fold in the presence of galactose in media without glucose, thus providing a major overproduction of the corresponding protein [39]. Overexpression of each Hsp104 homolog was confirmed by Western blotting (Supplemental Figure S1B). Overproduction of either Sp_Hsp104 or Sc_Hsp104 allowed survival of *S. cerevisiae* cells after a severe heat shock at 50°C, without any pre-treatment (Figure 3A, right panel). Hence, the *S. pombe* Hsp104 homolog complements thermotolerance defects of *Δhsp104 S. cerevisiae* cells as well as Sc_Hsp104.

To confirm that the thermotolerance supported by *Sp_hsp104^+^* is due to disaggregase activity of the protein, we performed luciferase reactivation assays [2]. As shown in Figure 3B, both Hsp104 homologs showed disaggregating activity when expressed under the control of the *Sc_HSP104* promoter. Under basal expression conditions, Sp_Hsp104 homolog is significantly less efficient than Sc_Hsp104 to reactivate luciferase. When overexpressed however, both Sp_Hsp104 and Sc_Hsp104 displayed a similar disaggregating activity. Together, these results indicate that the fission yeast Hsp104 homolog is a functional disaggregase in the budding yeast. Since Sp_Hsp104 provides thermotolerance to both *S. pombe* and *S. cerevisiae*, and is also able to reactivate bacterial luciferase, a protein without any close homolog in yeasts, these experiments indicate that Sp_Hsp104 is a disaggregase of broad specificity, as was shown for Sc_Hsp104 [2].

### The *S. pombe* Hsp104 homolog does not propagate nor cure the [*PSI^+^*] prion

**Figure 4:**
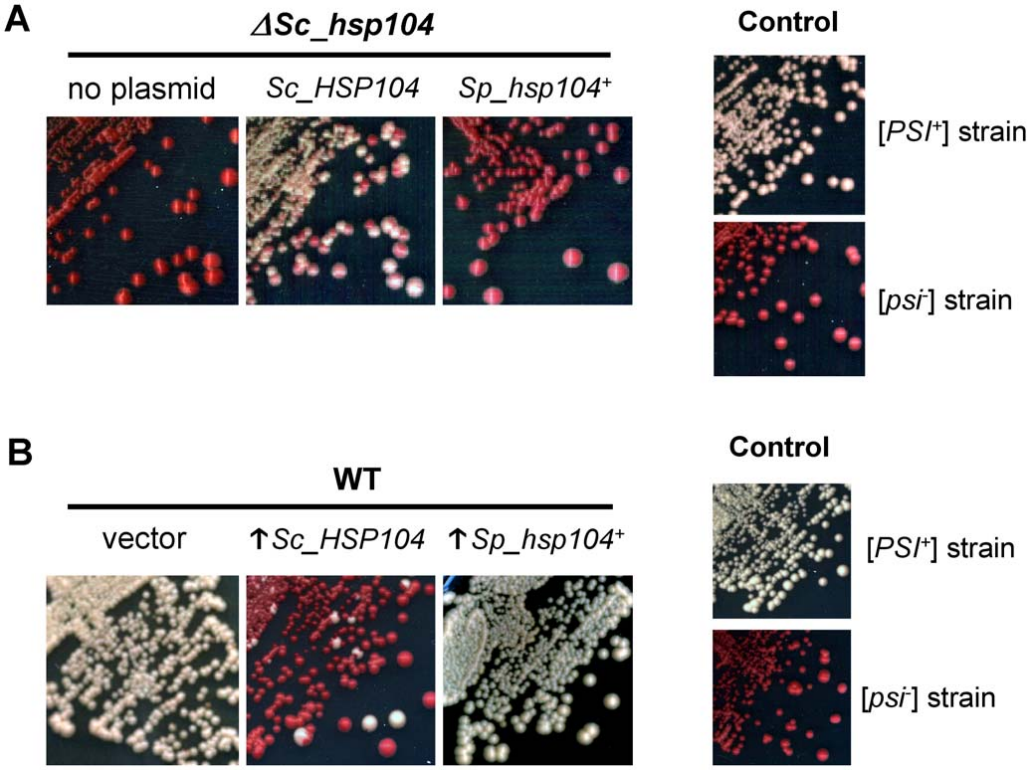
Sc*_*Hsp104 does not propagate or cure the *S. cerevisiae* [*PSI^+^*] prion. (A) Sp_Hsp104 cannot sustain [*PSI* ^+^] propagation. A [*PSI* ^+^] Δ*Sc_hsp104* strain complemented by a plasmidic *Sc_HSP104* gene (YJW532) was transformed with an empty vector or with a plasmid expressing *Sp_hsp104^+^* under the control of the endogenous *Sc_HSP104* promoter. After shuffling of the *Sc*_*HSP104*-encoding plasmid, cells were streaked on YPD¼ to test the maintenance of [*PSI* ^+^]. Control strains show the expected white color of [*PSI* ^+^] cells (Ψ-74-D694) and the red color of [*psi* ^−^] cells (74-D694). (B) Overexpression of Sp_Hsp104 does not cure [*PSI* ^+^]. A [*PSI* ^+^] strain (Ψ-74-D694) was transformed with an empty vector or with plasmids overexpressing (↑) either *Sc_HSP104* or *Sp_hsp104^+^* under the control of the *GAL1* promoter. Cells were streaked on YPD¼ to assess the appearance of red colonies, which indicates the curing of [*PSI* ^+^]. [*PSI* ^+^] (Ψ-74-D694) and [*psi* ^−^] (74-D694) strains are shown as color controls.

Another well-known function of Sc_Hsp104 is the propagation of *S. cerevisiae* prions [40]. Since propagation of [*PSI^+^*] requires the disaggregase activity of Hsp104, we wondered if the low activity of Sp_Hsp104 in *S. cerevisiae* would be sufficient to propagate [*PSI^+^*]. Because [*PSI^+^*] cannot be maintained in the absence of Sc_Hsp104, we used a plasmid-shuffling strategy to evaluate the ability of Sp_Hsp104 to maintain this yeast prion. To this end, the *Sp_hsp104^+^*-encoding plasmid was transformed into a [*PSI^+^*] *ΔSc_hsp104* strain (YJW532) complemented by an episomal copy of *Sc_HSP104* [28]. Simultaneous expression of both Hsp104 homologs did not affect the propagation of [*PSI^+^*], suggesting that Sp_Hsp104 does not have a dominant negative effect on the endogenous protein (not shown). Next, we shuffled the *Sc_HSP104*-encoding plasmid with 5-FOA. Loss of Sc_Hsp104 was confirmed by auxotrophy on selective media and Western blotting using monoclonal antibodies specifically recognizing Sc_Hsp104 (not shown). After streaking on YPD¼ medium, the control strain expressing Sc_Hsp104 efficiently propagated the [*PSI^+^*] prion, as observed by the white color of the colonies. In contrast, all the cells bearing the *Sp_hsp104^+^*-encoding plasmid were undistinguishable from the [*psi^−^*] strain (Figure 4A). Thus, *Sp_hsp104^+^* is unable to maintain the [*PSI^+^*] prion when expressed under the control of the endogenous *Sc_HSP104* promoter. Overexpression of *Sp_hsp104^+^* under the control of the *GAL1* promoter was also tested, but failed to sustain [*PSI^+^*] propagation in absence of Sc_Hsp104 (Supplemental Figure S2). This demonstrates that Sp_Hsp104 is unable to propagate [*PSI^+^*] and/or that interaction with species-specific cofactors are required for this activity.

Next, we assessed whether Sp_Hsp104 overexpression is able to cure [*PSI^+^*]. To this end, we transformed galactose-inducible plasmids encoding *Sp_hsp104^+^* or *Sc_HSP104* in [*PSI^+^*] cells. As expected, overexpression of Sc_Hsp104 cured the [*PSI^+^*] prion from most of the cells, resulting in the appearance of [*psi-*] colonies exhibiting the typical red color (Figure 4B). In contrast, heterologous overexpression of Sp_Hsp104 failed to cure [*PSI^+^*], as observed by the white color of colonies. Therefore, despite its disaggregating activity, Sp_Hsp104 is unable to block [*PSI^+^*] propagation when overexpressed.

### The CTD of Sc_Hsp104 is crucial for the curing of [*PSI^+^*]

**Figure 5:**
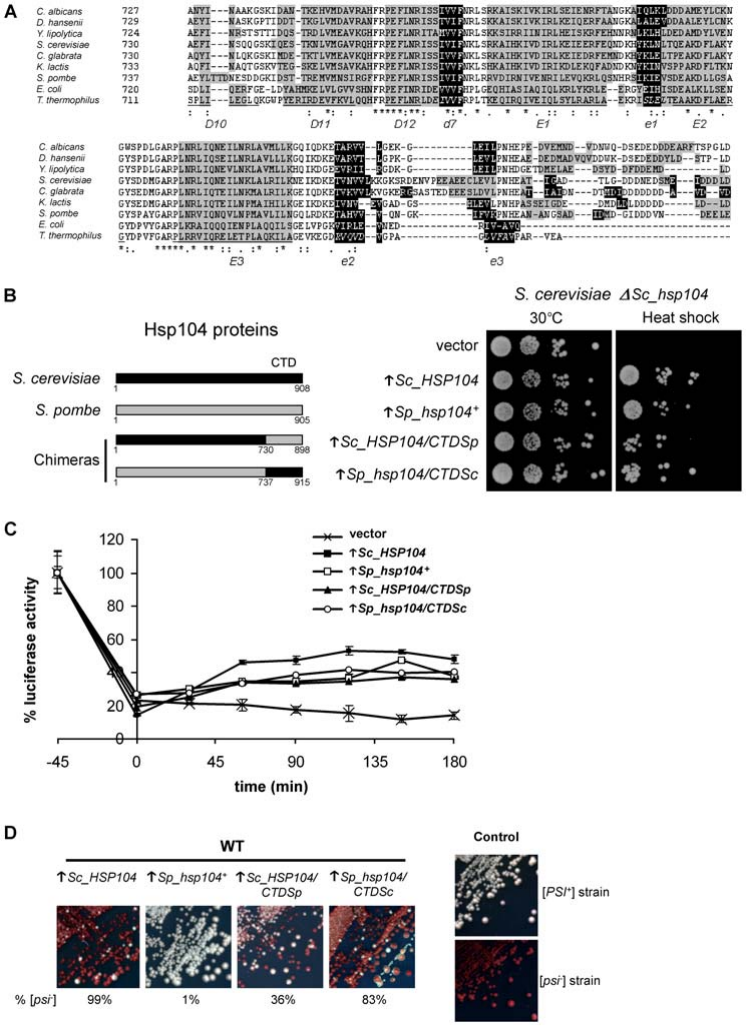
Involvement of the CTD of Sc_Hsp104 in [*PSI* ^+^] curing. (A) Analysis of the CTD of Hsp104 homologs. The sequence of seven eukaryotic Hsp104 and two bacterial ClpB homologs were aligned using the Clustal W2 program [69]. Identical (*), conserved (:) and semi-conserved (.) residues are indicated. Predicted alpha helixes are shaded in gray and beta strands are shaded in black. Structural elements determined by crystallography of *Thermus thermophilus* ClpB are also indicated for comparison [41]. Alpha helixes are underlined and beta strands are in *italic*. (B) Heterologous complementation of thermotolerance by chimeric Hsp104 proteins. Hsp104 chimeras were created by interchanging the CTD of Sc_Hsp104 (amino acids 731–908) with the CTD of Sp_Hsp104 (amino acids 738–905) (left panel). Δ*hsp104 S. cerevisiae* cells (SL303a) bearing an empty vector or overexpressing either *Sc_HSP104*, *Sp_hsp104^+^*, *Sc_HSP104/CTDSp* or *Sp_hsp104/CTDSc* under the control of the *GAL1* promoter were submitted to a severe heat shock of 50°C for 20 minutes (right panel). Cells were then briefly cooled on ice, serially diluted (10^−1^, 10^−2^, 10^−3^, 10^−4^), spotted on minimal media and incubated for 5 days at 30°C. Results are representative of three independent experiments. (C) Luciferase reactivation assay. Δ*hsp104 S. cerevisiae* cells (SL303a) expressing bacterial luciferase and an Hsp104 homolog or a chimera were submitted to a heat shock of 46°C for 45 minutes. Reactivation was assessed by measuring luciferase activity at the indicated time points and reporting the value on the initial activity (100%). Reactivation curves of cells bearing an empty vector (X) or overexpressing (↑) *Sc_HSP104* (▪), *Sp_hsp104^+^* (□), *Sc_HSP104/CTDSp* (▴) or *Sp_hsp104/CTDSc* (○) are indicated. Each point is the mean±standard deviation of three independent measures. (D) Curing of [*PSI* ^+^] by Hsp104 chimeras. A [*PSI* ^+^] strain (Ψ-74-D694) was transformed with an empty vector or with plasmids overexpressing (↑) *Sc_HSP104*, *Sp_hsp104^+^*, *Sc_HSP104/CTDSp* or *Sp_hsp104/CTDSc*. Cells were streaked on YPD¼ to assess the appearance of red colonies, which indicates the curing of [*PSI* ^+^]. The rate of [*PSI* ^+^] curing (% [*psi* ^−^]) was estimated on ¼YPD on at least 500 colonies from two different assays. [*PSI* ^+^] (Ψ-74-D694) and [*psi* ^−^] (74-D694) strains are shown as color controls.

Sp_Hsp104 confers thermotolerance in both *S. pombe* and *S. cerevisiae*. However, unlike Sc_Hsp104, the *S. pombe* homolog is unable to propagate [*PSI^+^*] in *Δhsp104* cells or to cure this prion by overexpression. Because the amino-acid sequences of Sp_Hsp104 and Sc_Hsp104 are not 100% identical (Figure 1A), some structural determinants must account for the inability of the *S. pombe* chaperone to propagate [*PSI^+^*]. To identify the less conserved structures between Sp_Hsp104 and Sc_Hsp104, we used the structural data obtained from ClpB [41] and Sc_Hsp104 [42] and a program for the prediction of secondary structures [43]. According to this analysis, Sc_Hsp104 and Sp_Hsp104 exhibit the least conserved secondary structure in the last segment of their CTD (Figure 5A). For instance, even though the NTD has a low level of amino acid identity (34% against 43% for the CTD), we were not able to identify any obvious secondary structure difference between the NTD of Sc_Hsp104 and that of Sp_Hsp104. On the other hand, the CTD has a very divergent region at the most C-terminal part of all Hsp104 homologs (Figure 5A). Further sequence alignments and secondary structure predictions confirmed that the CTD is the least conserved region between all ClpB/Hsp100 homologs that we analyzed.

Because Sp_Hsp104 and Sc_Hsp104 exhibit significant differences in the primary and secondary structures of their CTD, we investigated the role of this domain in [*PSI^+^*] propagation. As described in Figure 1A, we defined the CTDs of Sc_Hsp104 and Sp_Hsp104 by the region encompassing the amino acids 731–908 and 738–905, respectively. Using homologous recombination, we modified the coding sequence of both Sc_Hsp104 and Sp_Hsp104 by interchanging their CTD. These chimeric genes were named *Sc_HSP104/CTDSp* and *Sp_hsp104/CTDSc*. The modified genes were cloned into an *S. cerevisiae* overexpression vector and transformed into [*PSI* ^+^] cells. We confirmed that these plasmid-encoded chimeras (Sp_Hsp104/CTDSc and Sc_Hsp104/CTDSp) were expressed by Western blotting (Supplemental Figure S1C). Importantly, we confirmed that these overexpression chimeras were active in thermotolerance assays and retained disaggregase activity (Figure 5B and C).

Strikingly, the Sp_Hsp104/CTDSc chimera gained the ability to cure [*PSI* ^+^] (Figure 5D) without increasing its disaggregase activity (Figure 5C). This could be explained if the CTD from *S. cerevisiae* confers the ability to interact with [*PSI* ^+^] or if the chimeric chaperone has a dominant negative effect on the endogenous Sc_Hsp104. On the other hand, the Sc_Hsp104/CTDSp chimera was less efficient than WT Sc_Hsp104 to cure [*PSI* ^+^] (Figure 5D). This could be due to the lower protein level of Sc_Hsp104/CTDSp as compared to the WT Sc_Hsp104 and the Sp_Hsp104/CTDSc chimera (Supplemental Figure S1C). Alternatively, the CTD of Sp_Hsp104 could interfere with the ability of Hsp104 to interact with [*PSI* ^+^]. In any case, these results indicate that the CTD of Sc_Hsp104 plays an important role in the propagation of [*PSI* ^+^].

## Discussion

In this study we have characterized Sp_Hsp104, the fission yeast homolog of the Hsp104 chaperone. We demonstrated that the heat-inducible *Sp_hsp104^+^* gene is required for the acquisition of thermotolerance in *S. pombe* and that the Sp_Hsp104 protein is a functional disaggregase in the budding yeast. Nevertheless, unlike its *S. cerevisiae* counterpart, Sp_Hsp104 is unable to maintain the [*PSI^+^*] prion in *Δhsp104* cells and its overexpression does not cure [*PSI^+^*]. Interestingly, a chimera in which the CTD of Sp_Hsp104 was replaced with the corresponding domain of Sc_Hsp104 gained the ability to cure [*PSI^+^*].

Thermotolerance is a prime example of adaptation to environmental changes. In S. cerevisiae, specific heat-shock proteins and stress-activated response pathways are crucial for this process. In fission yeast, thermotolerance was originally linked to the metabolism of trehalose, a reserve sugar acting as a chemical chaperone [29]. Synergic effects between trehalose metabolism and Hsp104 were discovered in the budding yeast, suggesting a conserved thermotolerance pathway [44], [45]. Nevertheless, some differences were also observed between these two yeast species. For instance, the small heat shock protein Hsp16 was linked to thermotolerance in nuclear mRNA export in S. pombe, whereas the S. cerevisiae homolog of this protein, Hsp26, was shown to be dispensable for thermotolerance [46], [47]. Hence, it remained possible that each yeast species possesses specific chaperone machineries devoted to thermotolerance. Here we show that like in S. cerevisiae and C. albicans [28], the fission yeast Hsp104 exhibits disaggregating activity and is essential for thermotolerance. This suggests that Hsp104 has a conserved thermotolerance function in fungi.

Sc_Hsp104 is involved in the propagation of S. cerevisiae prions. Indeed, among the 24 yeast proteins containing a prion-forming domain recently identified in S. cerevisiae, only one was independent of Sc_Hsp104 for its propagation [48]. Because Hsp104 homologs from S. pombe and S. cerevisiae share a high level of sequence identity and are both functional disaggregases, we were surprised that Sp_Hsp104 failed to propagate or cure [PSI^+^]. Sp_Hsp104 confers thermotolerance in budding yeast when expressed under the control of the heat-inducible Sc_HSP104 promoter or under GAL1 overexpression. However, whereas the level of luciferase reactivation by Sp_Hsp104 was close to that of Sc_Hsp104 when overexpressed, Sp_Hsp104 was less efficient when expressed under the control of the Sc_HSP104 promoter on a centromeric plasmid. Therefore, the differences in disaggregating activities of the Hsp104 homologs could be, at least in part, responsible for the inability of Sp_Hsp104 to propagate and cure [PSI^+^].

Our analysis of the protein sequence of Hsp104 homologs prompted us to investigate the possible involvement of the CTD in [PSI^+^] propagation. Intriguingly, we found that a chimeric Sp_Hsp104 protein bearing the CTD from S. cerevisiae gained the ability to cure [PSI^+^]. This suggests that the CTD of Sc_Hsp104 modulates its prion-curing activity. Four non-exclusive possibilities may explain this gain of activity. First, the CTD of Hsp104 could be involved in [PSI^+^] recognition or binding. Indeed, previous studies proposed that the acidic C-terminal region of Hsp104 acts as a substrate-binding determinant for prion recognition [7]. Secondly, the CTD of Hsp104 could be involved in the recruitment of co-factors required for prion propagation. For instance, the Hsp90 co-factors Sti1p and Sgt2p interact with Hsp104 through the C-terminal tetratricopeptide-repeat (TPR)-like motif [49], [50]. A third possibility could be that the CTD of Sc_Hsp104 specifically increases the prion-propagation activity of the disaggregase without affecting its ability to untangle heat-aggregated proteins. Supporting this idea, the unstructured polypeptide poly-L-lysine was shown to stimulate the ATPase activity of both Sc_Hsp104 and ClpB by binding to the CTD region [51]. Finally, it is also possible that the curing of [PSI^+^] by overexpression of Sp_Hsp104/CTDSc is due to a dominant-negative effect of this chimera by interfering with the endogenous WT Sc_Hsp104 in the replication of [PSI^+^]. For instance, the CTD of the Sp_Hsp104/CTDSc chimera might interact with [PSI^+^] without promoting further replication of the prion.

Recently, Tipton et al. addressed the function of each Hsp104 domain by constructing chimeras with ClpB [13]. In contrast with our study, they concluded that the CTD of Hsp104 was dispensable for thermotolerance, disaggregation and prion propagation. Interestingly, a chimeric Hsp104 protein bearing the C-terminal end of ClpB was as efficient as the WT protein to propagate [PSI^+^]. ClpB lacks most of the amino acids corresponding to the CTD of Hsp104 (Figure 5A). Thus, it is unlikely that the Hsp104-ClpB chimera interacts with any Hsp104 CTD-specific binding factor. However, the chimera created by Tipton et al. also contained the NBD2 of ClpB. Unlike the NBD2 from S. cerevisiae, which has poor ATPase activity [27], the NBD2 of ClpB exhibits ATPase activity on its own [52]. Hence, it remains possible that the NBD2 of ClpB synergistically increases the prion-propagation activity of Hsp104. As such, the CTD of Hsp104 could be dispensable for prion propagation by Hsp104-ClpB because this chimera is a more efficient disaggregase than the WT Hsp104. On the other hand, Sp_Hsp104, which has a lower disaggregase activity in S. cerevisiae (Figure 3B), could require the CTD of Sc_Hsp104 to stimulate its ATPase activity in order to cure [PSI^+^]. This being said, although our CTD chimeras revealed the importance of this domain in prion curing, we cannot exclude that other structural differences between the fission and budding yeast homologs also account for the inability of Sp_Hsp104 to propagate [PSI^+^]. For instance, the NTD domain of Sc_Hsp104 was shown to be required for the curing of [PSI^+^] [25], and our sequence analysis revealed some disparities between Sp_Hsp104 and Sc_Hsp104 in this region. According to cryo-EM structural studies, both N- and C-terminal domains cap the cavity of the hexameric Hsp104 ring [42]. Therefore, N-terminal contributions to prion disaggregation activity might explain why the Sc_Hsp104/CTDSp chimera cures [PSI^+^] despite bearing the CTD from Sp_Hsp104.

Whereas Sp_Hsp104 is unable to propagate the [PSI^+^] in S. cerevisiae, does it assist in the propagation of prions in S. pombe? To date no prions have been identified in S. pombe. One prion-like element, [cif], was reported [53], [54], but its molecular nature remains to be elucidated. The knockout of Sp_hsp104^+^ had no effect on the maintenance of [cif], indicating that Sp_Hsp104 is not involved in the propagation of this epigenetic element (not shown). Since one of the 24 prions reported in S. cerevisiae is independent of Sc_Hsp104 for replication [48], it remains possible that some yet to be identified fission yeast prions require Sp_Hsp104 for their inheritance. Interestingly, genome-wide analyses showed that proteins bearing Q/N-rich regions characteristic of most yeast prions are almost inexistent in S. pombe, in contrast to S. cerevisiae [55], [56]. One can thus envision that this proteome unusually low in aggregation-prone proteins has favoured a divergent evolution for Sp_Hsp104, thus making it less efficient to untangle aggregates of Q/N-rich proteins.

In conclusion, our research demonstrates that Sp_Hsp104 is the first wild-type yeast AAA+ protein able to complement thermotolerance in *S. cerevisiae* but unable to propagate [*PSI^+^*]. Hence, the fission yeast Hsp104 could be used in further studies to discriminate the molecular requirements of prion propagation from those responsible of disaggregation of heat-aggregated proteins.

## Materials and Methods

### Strains and media

Yeast strains used in this study are listed in Table 1. Unless otherwise indicated, *S. pombe* strains were grown at 30°C in Edinburgh minimal medium (MM) supplemented with the required nutrients [57]. For growth of the various *S. cerevisiae* strains, standard growth media were used, and cells were routinely cultured at 30°C as previously described [58]. To ensure plasmid retention, transformed cells were grown on a selective synthetic media (SD, also called YNBD) containing all necessary supplements (Sigma). The [*PSI^+^*]-mediated suppression of the *ade1*-*14* marker was routinely assessed by the color of colonies formed on YPD¼ medium (YEPD with 2.5 g/L of yeast extract rather than 10 g/L) and confirmed on SD-adenine defined medium supplemented with 2.5% (vol/vol) YEPD. The *GAL1* promoter was induced by incubating cells on SDGal (SD containing 20 g of galactose/L instead of glucose as the carbon source) after preliminary growth in SDLG (YEPD containing 3% of lactate and 3% of glycerol instead of glucose) to eliminate all glucose from the liquid medium [59].

**Table 1:** Yeast strains used in this study.

Species	Strain	Genotype	Reference
*S. pombe*	SP3220 (WT)	*h* ^−^ *his3-D1 ade6-M216 ura4-D18 leu1-32* Δ*cnx1::his3*+pREP41*cnx1* ^+^	Elagoz *et al.* (1999) [70]
*S. pombe*	SP12422 (Δ*Sp_hsp104*)	SP3220 Δ*hsp104::neo^R^*	This study
*S. cerevisiae*	W303a (WT)	*MAT*a *leu2-3,112 trp1-1 ura3-1 ade2-1 his3-11,15 lys2*Δ *can1-100*	Thomas *et al.* (1989) [71]
*S. cerevisiae*	SL303a (Δ*Sc_hsp104*)	W303a Δ*hsp104::LEU2*	Sanchez *et al.* (1990) [1]
*S. cerevisiae*	YJW532 [*PSI^+^*]	*MAT*a *ade1*-*14 his3*-*11*,*15 leu2*-*3*,*112 ura3*-*1 trp1*-*1 can1*-*100 hsp104*::*HIS3*+pRS316-*Sc_HSP104*	Zenthon *et al.* (2006) [28]
*S. cerevisiae*	Ψ-74-D694 [*PSI^+^*]	*MAT*a *ade1-14 his3*Δ*-200 leu2-3 trp1-289 ura3-52*	Chernoff *et al.* (1995) [15]
*S. cerevisiae*	74-D694 [*psi*-]	*MAT*a *ade1-14 his3*Δ*-200 leu2-3 trp1-289 ura3-52*	Chernoff *et al.* (1995) [15]

### Identification and deletion of the *S. pombe hsp104^+^* gene

Using the *S. cerevisiae* Hsp104 (YLL026W) amino acid sequence, the *S. pombe* genome database was searched with the BLASTP alignment program [60]. A single sequence at locus NP_596503 encoding the hypothetical protein SPBC16D10.08c showed significant sequence identity (52%) to the Sc*_* Hsp104 protein sequence. The deletion of *Sp_hsp104^+^* was carried out by the method described in Krawchuk and Wahls [61]. Using the pFA6a-KanMX6 plasmid [62] as a template, the neomycin resistance gene was amplified with the Neo_FW and Neo_REV primers (Table 2). *Sp_hsp104^+^* was amplified with its flanking regions from fission yeast genomic DNA using the primers Sp_Hsp104_FL_FW and Sp_Hsp104_FL_REV and cloned into the pCR-XL-TOPO vector. The coding region of *Sp_hsp104* ^+^ was extracted by a *Age*I/*Pac*I digestion and replaced with the neomycin resistance gene. The *Sp_hsp104^+^* knockout cassette was extracted from pCR-XL-TOPO by *Xho*I digestion and transformed into strain SP3220. Southern blot analyses were performed to confirm the correct and unique insertion of the cassette in the *S. pombe* genome using standard methods [57], [63] (not shown).

**Table 2:** Oligonucleotides used in this study.

Name	Sequence*	Restriction site
Neo_FW	5′-C*ACCGGT*CCGGGTTAATTAA-3′	*Age*I
Neo_REV	5′-CC*TTAATTAA*CGAGCTCGTTTAAACTGG-3′	*Pac*I
Sp_Hsp104_FL_FW	5′-*CTCGAG*CAACCTCTTCATCCTCAG-3′	*Xho*I
Sp_Hsp104_FL_REV	5′-*CTCGAG*CCATATTAGCTGCTACCG-3′	*Xho*I
Sp_Hsp104_REC_FW	5′-CAAAGAAAAAAGAAATCAACTACACGTACCATAAAATATACAGAATATATGGCTGATTATCCTTTTACTGAC-3′	
Sp_Hsp104_REC_REV	5′-AGAGTTCCAATTCTTCTTGCAATGAAGCTTCCTTCTGCCTAGCTAACTTTATTCCAATTCTTCATCATTAAC-3′	
Sc_Hsp104_UTR_FW	5′-ATACATATCCATATCTAATCTTACTTATATGTTGTGGAAATGTAAAGAGC*GCGGCCGC*ATCGATTCAAAGGCGTTATTCAGC-3′	***NotI***
Sc_Hsp104_UTR_REV	5′-CATATATTCTGTATATTTTATGGTACGTG-3′	
Sp_Hsp104_CTD_FW	5′-AAGACGATCGACTGTTCCAATTGTATTGTCATCATGACTTCCAATCTAGGTGCTGAATACTTGACAACAGACAATGAGTCT-3′	
Sp_Hsp104_CTD_REV	5′-TTATATTACTGATTCTTGTTCGAAAGTTTTTAAAAATCACACTATATTAAATTATTCCAATTCTTCATCATTAACATCGTC-3′	
Sc_Hsp104_CTD_FW	5′-CAGGTTGTTGATGCCAAGAATGCTGTTATCATTATGACTTCTAACTTGGGCGCTGAATTTATCAATTCTCAACAAGGATCA-3′	
Sc_Hsp104_CTD_REV	5′-ATATTACTGATTCTTGTTCGAAAGTTTTTAAAAATCACACTATATTAAACTTTAATCTAGGTCATCATCAATTTCCATACT-3′	

* Restriction sites are in italic.

### Plasmid constructions and transformation

Plasmids used in this study are described in Table 3. All plasmids expressing the *S. cerevisiae HSP104* gene are from the Susan Lindquist lab. Plasmids expressing the *S. pombe hsp104* ^+^ gene were created by *in vivo* recombination in *S. cerevisiae*. First, the *Sp_hsp104^+^* gene was amplified using the Sp_Hsp104_REC_FW and Sp_Hsp104_REC_REV primers to add 50 bp corresponding to the flanking regions of the pYSGAL-*Sc_HSP104* plasmid on either side of the coding sequence. The *Bgl*II-linearized pYSGAL-*Sc_HSP104* vector and the PCR amplification of *Sp_hsp104^+^* were then transformed into W303a competent *S. cerevisiae* cells with the specifications described in Knop *et al.* (1999) [64] for recombination. After selection for *URA3*, the pYSGAL-*Sp_hsp104^+^* plasmid was extracted using the lyticase extraction protocol of Ling *et al.* (1995) [65]. For creation of the pRS315 and pRS316 plasmids, the *GAL1* promoter of the pYSGAL-*Sp_hsp104^+^* plasmid was replaced by 640 pb of the genomic 5′UTR of *S. cerevisiae HSP104*. The 5′UTR was amplified using the Sc_HSP104_UTR_FW and Sc_HSP104_UTR_REV primers. The *Sp_hsp104^+^* gene under the control of the endogenous *S. cerevisiae* promoter was then extracted by *Not*I digestion and cloned into pRS315 and pRS316. The chimeric genes were constructed using the same recombination approach using PCR amplification of the CTDs of each gene. The CTD from *S. pombe* Hsp104 was amplified using the Sp_Hsp104_CTD_FW and Sp_Hsp104_CTD_REV primers, while the CTD from *S. cerevisiae* Hsp104 was amplified using the Sc_Hsp104_CTD_FW and Sc_Hsp104_CTD_REV primers. All PCR amplifications were performed with the Phusion™ High-Fidelity DNA Polymerase (NEB, Ipswich, MA, USA). All plasmid constructions were verified by standard sequencing methods (IRIC genomic platform, Montréal, Canada). In addition, all plasmids were tested for protein expression by immunoblotting with appropriate antibodies. DNA transformations into *S. pombe* and *S. cerevisiae* cells were performed by the polyethylenglycol (PEG)-lithium acetate procedure [66].

**Table 3:** Plasmids used in this study.

Plasmid	Features	Source
pRS316	pBluescript-based centromeric yeast expression vector with *URA3* gene for selection	ATCC
pRS316-*Sc_HSP104*	pRS316 expressing the *HSP104* gene from *S. cerevisiae* under the control of the endogenous promoter (588 bp)	S. Lindquist Lab
pRS316-*Sp_hsp104^+^*	pRS316 expressing the *hsp104^+^* gene from *S. pombe* under the control of the endogenous *S. cerevisiae HSP104* promoter (588 bp)	This study
pRS315	pBluescript-based centromeric yeast expression vector with *LEU2* gene for selection	ATCC
pRS315-*Sp_hsp104^+^*	pRS315 expressing the *hsp104^+^* gene from *S. pombe* under the control of the endogenous *S. cerevisiae HSP104* promoter (588 bp)	This study
pGPD-*luxAB*(HIS)	p426GPD vector expressing a bacterial temperature-sensitive *Vibrio harveyi* luciferase	S. Lindquist Lab
pYSGAL	pRS316 with the galactose-inducible *GAL1* overexpression promoter	S. Lindquist Lab
pYSGAL-*Sc_HSP104*	pYSGAL overexpressing the *HSP104* gene from *S. cerevisiae*	S. Lindquist Lab
pYSGAL-*Sp_hsp104^+^*	pYSGAL overexpressing the *hsp104^+^* gene from *S. pombe*	This study
pYSGAL-*Sc_HSP104/CTDSp*	pYSGAL overexpressing a chimeric *HSP104* gene from *S. cerevisiae* (first 2190 bp) with the CTD of the *hsp104* ^+^ gene from *S. pombe* (last 507 bp)	This study
pYSGAL-*Sp_hsp104/CTDSc*	pYSGAL overexpressing a chimeric *hsp104* ^+^ gene from *S. pombe* (first 2211 bp) with the CTD of the *HSP104* gene from *S. cerevisiae* (last 537 bp)	This study

### Antibodies and immunoblotting

For the specific detection of Hsp104 from *S. cerevisiae*, we used the commercially available polyclonal rabbit antibodies directed against the last residues of Sc_Hsp104 (Stressgen) or the monoclonal antibodies described in Cashikar *et al.* (2002) [7], which specifically recognize the M domain of Sc_Hsp104. In contrast to a previous report [67], we were not able to detect Sp_Hsp104 with the commercial anti-Hsp104 antibodies. For the detection of Sp_Hsp104, we used antibodies raised against the whole recombinant His-tagged Sc_Hsp104 protein described in Tkach *et al.* (2004) [6]. These polyclonal antibodies were able to detect Sp_Hsp104 in a specific manner when used at a dilution of 1∶5000. To eliminate background, we pre-blotted these antibodies with an empty nitrocellulose membrane and we treated them with acetone powder of the Δ*Sp_hsp104* strain, as described in Sambrook *et al.* (1989) [63]. For Western blotting, standard immunoblotting procedures were used [68]. Bands were quantified using the Quantity One software (BioRad).

### Thermotolerance assay

Exponentially growing cells were adjusted to an OD_595_ of 0.5, serially diluted (10^−1^ to 10^−4^), spotted on solid media and grown for 5 days at 30°C. Heat shock was performed on exponentially growing cells adjusted to an OD_595_ of 0.5. Cells were pre-treated or not at 37°C for 1 hour, and then incubated with slight agitation at 50°C for 20 minutes and cooled on ice for 5 minutes. Cells were mixed by vortexing, serially diluted and subsequently spotted on the corresponding solid media.

### Luciferase reactivation assay

The luciferase reactivation assay was essentially performed as described in Zenthon *et al.* (2006) [28]. Briefly, the relevant strains were transformed with the plasmid pGPD-*luxAB*(HIS) (AddGene #1106), which expresses a temperature-sensitive *Vibrio harveyi* luciferase [2]. The transformed cells were grown to an OD_595_ of approximately 0.5. The luciferase activity was determined before treatment as a control. The culture was then transferred to 46°C, and after 30 minutes of incubation at this temperature, cycloheximide was added to a final concentration of 10 µg/mL. The culture was then incubated for further 15 minutes, after which the cell culture was transferred back to 25°C to allow the cells to recover. Cell samples were taken immediately to determine the level of luciferase activity and then collected every 30 to 45 minutes for up to 4 hours. The luciferase activity was determined by using 200 µL of cells plus 5 µL decylaldehyde (Sigma), and the resulting luminescence was immediately quantified using a Lumat LB 9507 luminometer (EG&G Berthold). Three independent samples were taken per time point.

## Supporting Information

Figure S1 **Expression and overexpression of Hsp104 homologs and chimeras** **(A)** Expression of Sc_Hsp104 and Sp_Hsp104 was verified by immunoblotting. Protein extracts from *S. cerevisiae Δhsp104* strains bearing an empty vector or expressing *Sc_HSP104* or *Sp_hsp104^+^* under the control of the endogenous *Sc_HSP104* promoter were separated by SDS-PAGE and immunoblotted using monoclonal anti-Hsp104 antibodies (left panel) or polyclonal antibodies raised against the full-length protein (right panel). The monoclonal antibodies specifically recognized the Sc_Hsp104 protein, while the polyclonal antibodies from Tkach and Gover (2004) were the only ones able to detect Sp_Hsp104, when concentrated at a dilution of 1∶5000. Immunoblotting of Pgk1p (phosphoglycerate kinase) is shown as a loading control. **(B)** Overexpression of Sc_Hsp104 and Sp_Hsp104 was verified by immunoblotting. Protein extracts from *S. cerevisiae Δhsp104* strains bearing an empty vector or overexpressing *Sc_HSP104* or *Sp_hsp104^+^* under the control of the *GAL1* promoter were separated by SDS-PAGE and immunoblotted using monoclonal anti-Hsp104 antibodies (left panel) or polyclonal antibodies raised against the full-length protein (right panel). Immunoblotting of Pgk1p is shown as a loading control. **(C)** Overexpression of Hsp104 chimeras was verified by immunoblotting. Protein extracts from *S. cerevisiae Δhsp104* strains bearing an empty vector or overexpressing either *Sc_HSP104*, *Sp_hsp104^+^*, *Sc_HSP104/CTDSp* or *Sp_hsp104/CTDSc* under the control of the *GAL1* promoter were separated by SDS-PAGE and immunoblotted using polyclonal antibodies directed against the CTD of Sc_Hsp104 (Stressgen, upper panel) or monoclonal anti-Hsp104 antibodies (middle panel). Immunoblotting of Pgk1p is shown as a loading control (lower panel).
(0.59 MB TIF)

Figure S2 **Overexpression of Sp_Hsp104 cannot sustain [PSI+] propagation** A [*PSI^+^*] *ΔSc_hsp104* strain complemented by a plasmidic *Sc_HSP104* gene (YJW532) was transformed with an empty vector or with a plasmid overexpressing *Sp_hsp104^+^* under the control of the *GAL1* promoter. After shuffling of the *Sc_HSP104*-encoding plasmid, cells were streaked on YPG1/4 to test the maintenance of [*PSI^+^*]. Control strains show the expected white color of [*PSI^+^*] cells (Ψ-74-D694) and the red color of [*psi^−^*] cells (74-D694)
(3.84 MB TIF)
