# Supplementary figures and images for: The Schizosaccharomyces pombe Hsp104 Disaggregase Is Unable to Propagate the [PSI +] Prion
Source: PLoS One. 2009 Sep 11;4(9):e6939. doi: 10.1371/journal.pone.0006939 (PMC2736384; doi:10.1371/journal.pone.0006939)

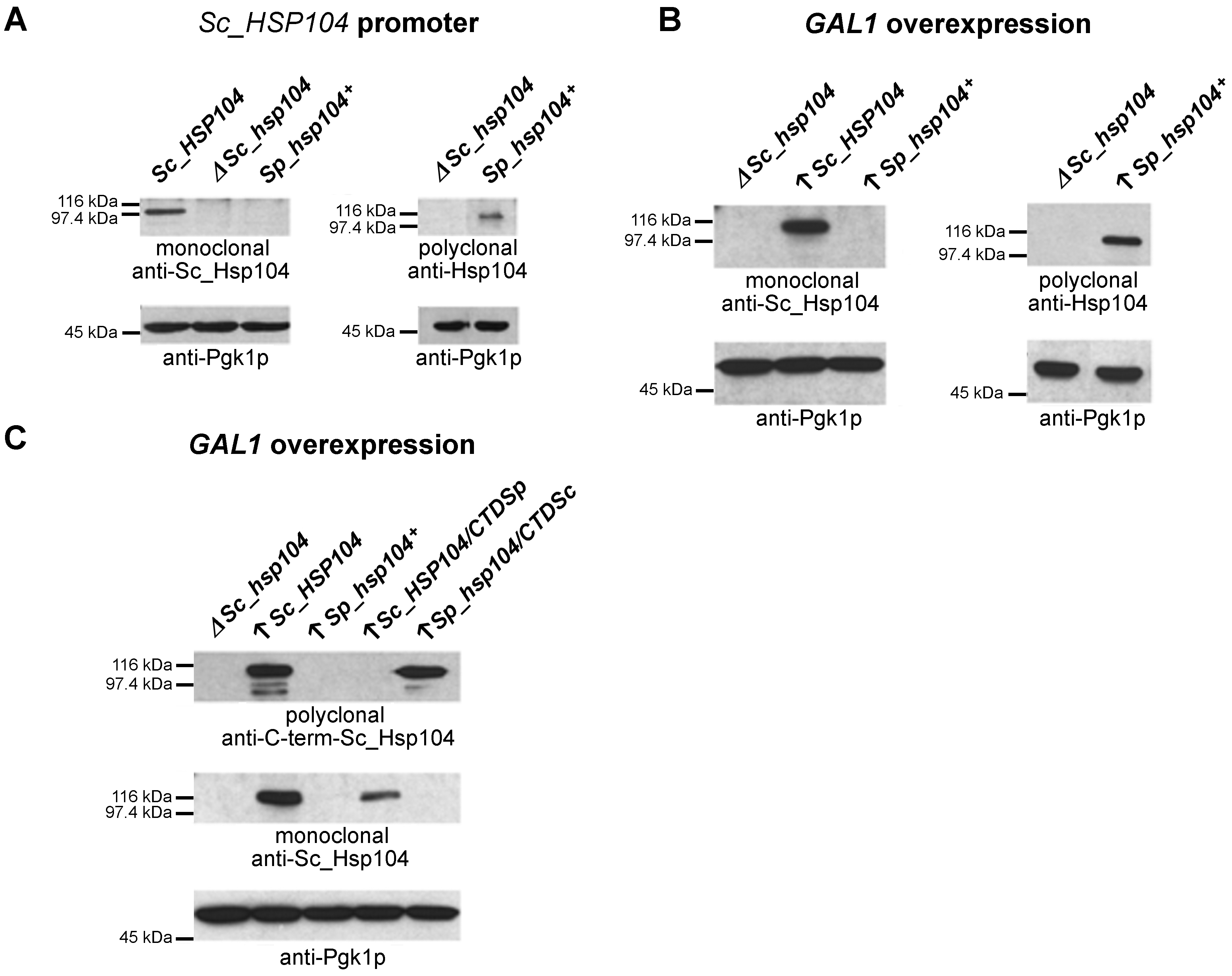

Supplement: Figure S1 — Expression and overexpression of Hsp104 homologs and chimeras (A) Expression of Sc_Hsp104 and Sp_Hsp104 was verified by immunoblotting. Protein extracts from S. cerevisiae Δhsp104 strains bearing an empty vector or expressing Sc_HSP104 or Sp_hsp104+ under the control of the endogenous Sc_HSP104 promoter were separated by SDS-PAGE and immunoblotted using monoclonal anti-Hsp104 antibodies (left panel) or polyclonal antibodies raised against the full-length protein (right panel). The monoclonal antibodies specifically recognized the Sc_Hsp104 protein, while the polyclonal antibodies from Tkach and Gover (2004) were the only ones able to detect Sp_Hsp104, when concentrated at a dilution of 1∶5000. Immunoblotting of Pgk1p (phosphoglycerate kinase) is shown as a loading control. (B) Overexpression of Sc_Hsp104 and Sp_Hsp104 was verified by immunoblotting. Protein extracts from S. cerevisiae Δhsp104 strains bearing an empty vector or overexpressing Sc_HSP104 or Sp_hsp104+ under the control of the GAL1 promoter were separated by SDS-PAGE and immunoblotted using monoclonal anti-Hsp104 antibodies (left panel) or polyclonal antibodies raised against the full-length protein (right panel). Immunoblotting of Pgk1p is shown as a loading control. (C) Overexpression of Hsp104 chimeras was verified by immunoblotting. Protein extracts from S. cerevisiae Δhsp104 strains bearing an empty vector or overexpressing either Sc_HSP104, Sp_hsp104+, Sc_HSP104/CTDSp or Sp_hsp104/CTDSc under the control of the GAL1 promoter were separated by SDS-PAGE and immunoblotted using polyclonal antibodies directed against the CTD of Sc_Hsp104 (Stressgen, upper panel) or monoclonal anti-Hsp104 antibodies (middle panel). Immunoblotting of Pgk1p is shown as a loading control (lower panel). (0.59 MB TIF) [file pone.0006939.s001.tif]

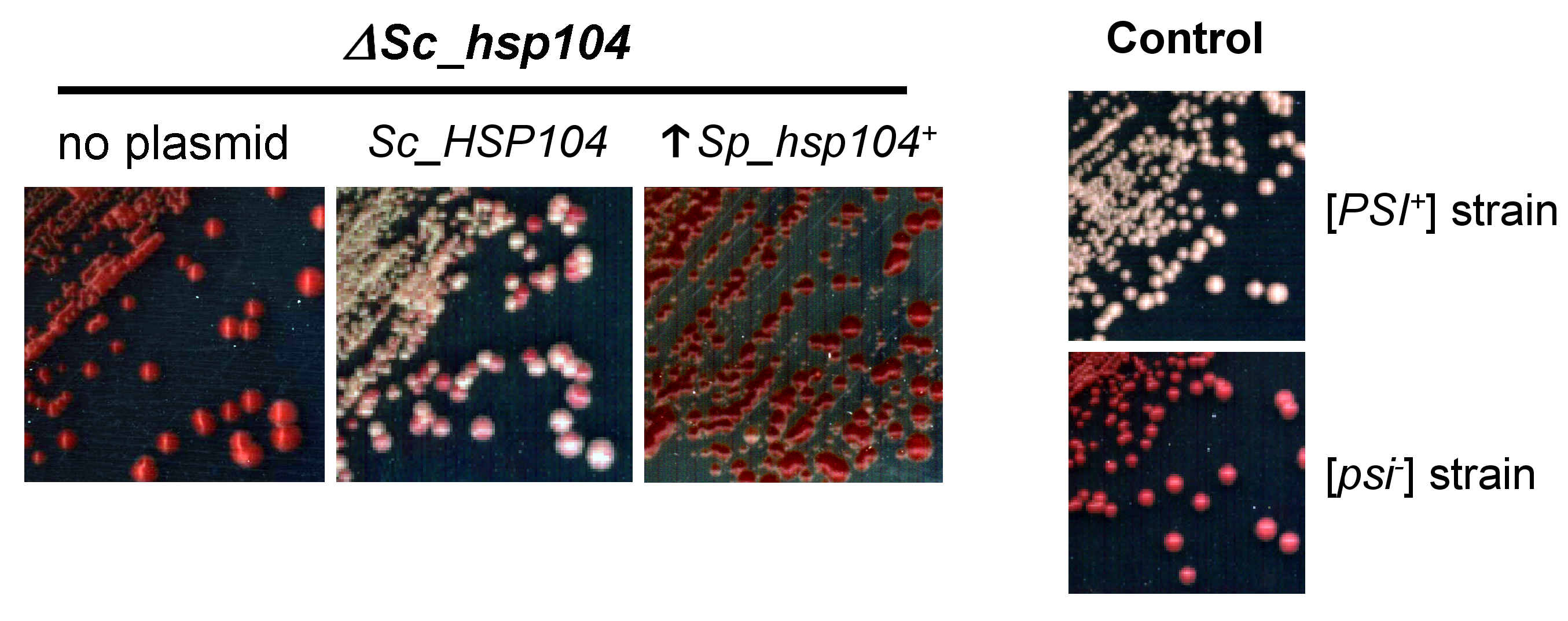

Supplement: Figure S2 — Overexpression of Sp_Hsp104 cannot sustain [PSI+] propagation A [PSI+] ΔSc_hsp104 strain complemented by a plasmidic Sc_HSP104 gene (YJW532) was transformed with an empty vector or with a plasmid overexpressing Sp_hsp104+ under the control of the GAL1 promoter. After shuffling of the Sc_HSP104-encoding plasmid, cells were streaked on YPG1/4 to test the maintenance of [PSI+]. Control strains show the expected white color of [PSI+] cells (Ψ-74-D694) and the red color of [psi−] cells (74-D694) (3.84 MB TIF) [file pone.0006939.s002.tif]
